# Dual GSK3β/SIRT1 modulators for Alzheimer’s: mechanisms, drug discovery and future perspectives

**DOI:** 10.3389/fphar.2025.1662241

**Published:** 2025-09-15

**Authors:** Afeez I. Kareem, Erika Kapp, Jacques Joubert, Xiaoqin Zou

**Affiliations:** ^1^ Department of Pharmaceutical Chemistry, School of Pharmacy, University of the Western Cape, Bellville, South Africa; ^2^ Department of Physics, University of Missouri, Columbia, MO, United States; ^3^ Department of Biochemistry, University of Missouri, Columbia, MO, United States; ^4^ Dalton Cardiovascular Research Center, University of Missouri, Columbia, MO, United States; ^5^ Institute for Data Science and Informatics, University of Missouri, Columbia, MO, United States

**Keywords:** Alzheimer’s disease, GSK3β inhibition, SIRT1 activation, tau hyperphosphorylation, dual-target drug design, polypharmacology, resveratrol, cheminformatics

## Abstract

Alzheimer’s disease (AD) remains without effective disease-modifying therapies, in part due to the limited efficacy of single-target approaches. Dual modulation of glycogen synthase kinase-3β (GSK3β), a key driver of tau hyperphosphorylation and amyloid-β (Aβ) production, and sirtuin-1 (SIRT1), a neuroprotective NAD^+^-dependent deacetylase, has emerged as a promising therapeutic strategy. This review explores the mechanistic rationale for concurrently inhibiting GSK3β and activating SIRT1 to disrupt AD’s pathological cascade while enhancing endogenous neuroprotective pathways. Natural compounds such as resveratrol, berberine, pterostilbene, and quercetin exhibit this dual activity and provide scaffolds for rational drug design. However, challenges related to target selectivity, blood-brain barrier penetration, and clinical translation persist. Advances in multi-target drug discovery, including pharmacophore hybridization, structure-based modelling, cheminformatics, nanoformulation and delivery strategies offer new avenues to overcome these hurdles. A dual GSK3β/SIRT1-targeting strategy exemplifies a systems-level approach to restoring neurophysiological balance and holds potential to achieve more effective, disease-modifying outcomes in AD.

## Introduction

Alzheimer’s disease is a multifactorial neurodegenerative disorder characterized by progressive cognitive decline and hallmark brain lesions: extracellular amyloid-β (Aβ) plaques and intracellular neurofibrillary tangles (NFTs) of hyperphosphorylated tau (Jellinger, 2022; Kaur et al., 2021). These lesions arise from a complex network of pathogenic processes, including protein aggregation, synaptic dysfunction, metabolic and mitochondrial disturbances, oxidative stress, and chronic neuroinflammation (Jurcău et al., 2022). Given this complexity, it is perhaps not surprising that traditional single-target therapies (e.g., drugs aiming only at Aβ production/clearance or only at tau kinases) have so far failed to produce disease-modifying effects in clinical trials. This shortcoming has spurred a paradigm shift toward multi-target therapeutic strategies that address multiple pathways of AD simultaneously (Benek et al., 2020). Multi-target directed ligands (MTDLs), single molecules designed to modulate two or more disease-relevant targets, are expected to achieve synergistic efficacy across the various mechanisms of AD while potentially reducing the need for polypharmacy and lowering off-target side effects (Badria et al., 2025). In other words, hitting several key nodes in the AD pathological network at once could yield more pronounced neuroprotection than one-dimensional approaches.

Within this multi-target therapeutic framework, one particularly promising strategy is the development of dual-acting compounds that both inhibit GSK3β and activate SIRT1. GSK3β is a serine/threonine kinase heavily implicated in tau hyperphosphorylation and other pathological processes in AD, whereas SIRT1 is an NAD^+^-dependent deacetylase that governs stress resistance, metabolic regulation, and protein homeostasis in neurons (Chauhan et al., 2022; Santos et al., 2023). A single compound capable of dampening GSK3β activity while boosting SIRT1 function could theoretically deliver a “one-two punch” against AD: concurrently reducing toxic tau phosphorylation and related neurodegeneration via GSK3β inhibition and enhancing neuronal survival pathways and proteostasis via SIRT1 activation. This review discusses the rationale behind targeting these two enzymes together, outlines strategies for designing such dual-function drugs, highlights examples of compounds that exhibit this dual activity along with their preclinical evidence and considers the challenges and future directions for translating this approach into a therapy.

## Rationale for multi-target approaches in Alzheimer’s treatment

Because AD’s pathology involves many interrelated processes, focusing on any single molecular target often fails to halt the disease (Tatulian, 2022). For instance, therapies aimed solely at reducing Aβ production or removing plaques do little to address tau pathology, neuroinflammation, or neuronal death (Nisbet and Götz, 2018). This may explain why numerous monotherapy trials (whether anti-amyloid or anti-tau) have shown limited clinical benefits despite successfully engaging their targets. In contrast, a multi-target approach, via combination therapies or, ideally, a single multi-functional agent, seeks to modulate several pathogenic pathways in parallel (Benek et al., 2020). By intervening at multiple points in AD’s pathogenic “web,” one hopes to achieve additive or synergistic effects on slowing neurodegeneration. For example, combining pro-cognitive cholinergic activity with anti-amyloid and anti-tau actions might preserve cognition more effectively than any one approach alone (Ailioaie et al., 2023; Cacabelos et al., 2016). Indeed, preclinical studies suggest that hitting multiple targets can produce more pronounced neuroprotection than one-dimensional treatments (Sousa et al., 2024).

The concept of multi-target directed ligands for AD has gained traction, with researchers designing hybrid molecules that merge pharmacophores for different targets into one compound (M. Singh et al., 2016; Sousa et al., 2024). Several such MTDLs have demonstrated enhanced efficacy in cell and animal models by simultaneously countering multiple neurotoxic processes (Katselou et al., 2014; Khan et al., 2025; Zaafar et al., 2024). One example is a *tacrine–pyrimidone* hybrid molecule that inhibits both acetylcholinesterase (AChE) and GSK3β. This single agent improved memory in mice better than an AChE inhibitor alone by virtue of its dual action (Yao et al., 2021). In general, rationally engineered dual-function compounds can retain potent activity at each intended target and confer broad neuroprotective effects in models. Clinically, a single polyfunctional drug is also attractive because it could reduce the pill burden and risk of drug–drug interactions for elderly AD patients (Salave et al., 2023).

**FIGURE 1:**
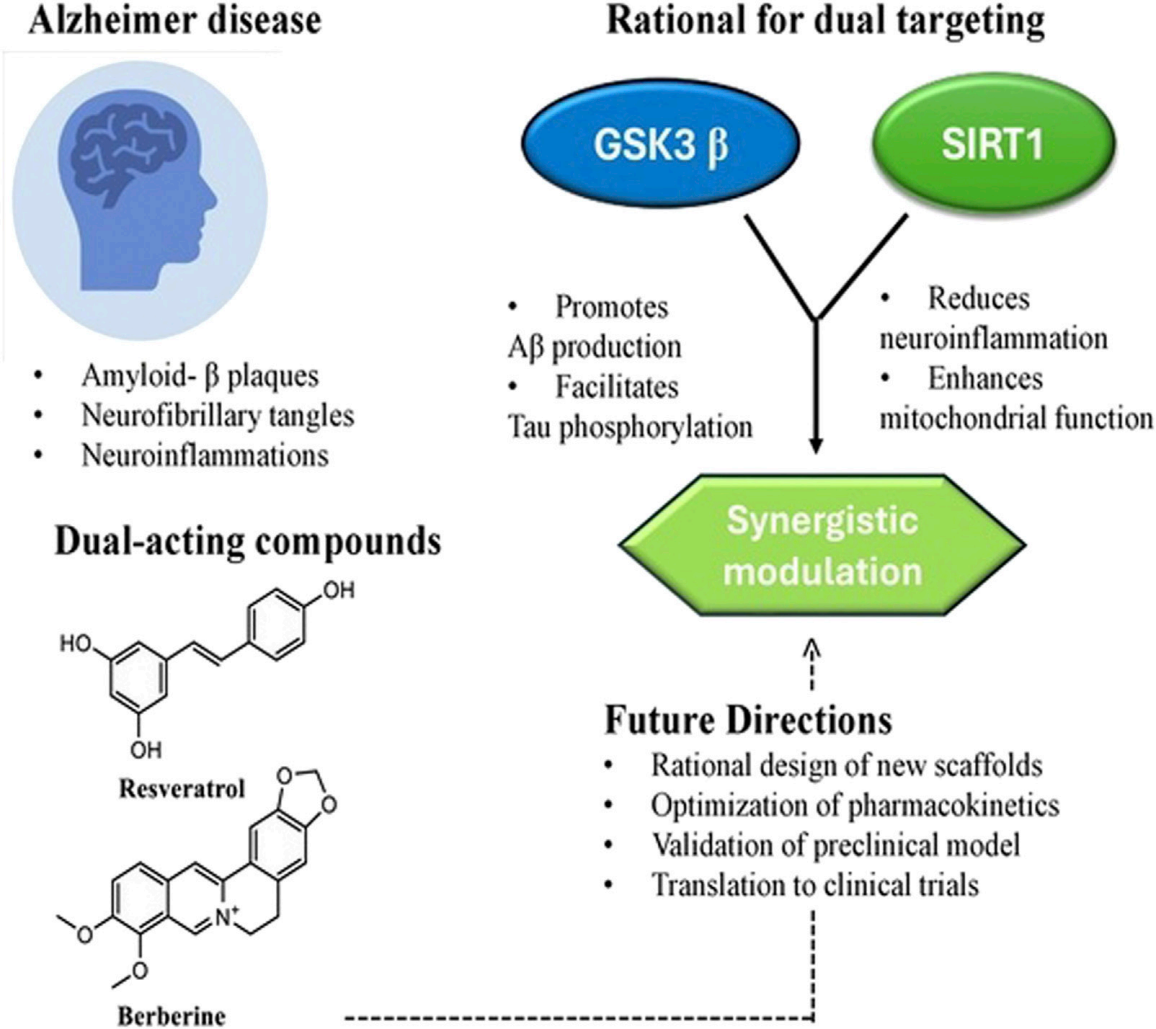
Dual targeting of GSK3β and SIRT1 in Alzheimer’s disease.

Why target GSK3β and SIRT1 together? This particular pair of targets is compelling because they sit on opposite sides of the neurodegenerative process (Figure 1). GSK3β overactivity drives several toxic features of AD especially tau pathology and associated synaptic dysfunction (Sayas and Ávila, 2021; Shareena et al., 2023), whereas SIRT1 activity promotes adaptive, protective responses in neurons enhancing protein clearance, stress resistance, and metabolic balance (Xu et al., 2018). In essence, inhibiting GSK3β addresses a key source of neuronal injury, while activating SIRT1 boosts the cell’s own defence and repair mechanisms. Therefore, a dual GSK3β inhibitor/SIRT1 activator is expected to simultaneously suppress a major pathological cascade (tau hyperphosphorylation and related toxicity) and amplify neuroprotective pathways such as non-amyloidogenic amyloid precursor protein (APP) processing and autophagy, yielding a synergistic therapeutic effect.

## Pathophysiological role of GSK3β in Alzheimer’s disease

GSK3β is a ubiquitously expressed serine/threonine kinase that, in the adult brain, plays normal roles in cellular signalling and plasticity (Lai et al., 2023). However, several evidence implicate aberrant overactivation of GSK3β as a key driver of AD pathogenesis (Hooper et al., 2008; Sayas and Ávila, 2021). GSK3β is one of the primary kinases responsible for tau protein phosphorylation. Under pathological conditions, excessive GSK3β activity leads to hyperphosphorylation of tau, causing tau to dissociate from microtubules and aggregate into neurofibrillary tangles (Sayas and Ávila, 2021). In transgenic models, overexpressing or chronically activating GSK3β induces tau pathology and cognitive deficits, whereas pharmacologically inhibiting GSK3β prevents those changes (Arciniegas Ruiz and Eldar-Finkelman, 2022). This evidence underpins the “GSK3 hypothesis” of AD, which posits that overactive GSK3 (particularly the β isoform) is a central contributor to AD pathology (Hooper et al., 2008).

Beyond tau, GSK3β can influence other aspects of AD (Abyadeh et al., 2024). It has been shown to enhance Aβ production, for example, by upregulating β-secretase and modulating APP processing, and to promote synaptic dysfunction (Sayas and Ávila, 2021). GSK3β also exacerbates neuroinflammation via activation of pro-inflammatory transcription factors and can trigger apoptotic pathways in neurons (Lai et al., 2023). Consistent with these widespread effects, various GSK3 inhibitors have demonstrated neuroprotective benefits in AD models (Karati et al., 2025). Treatment with GSK3 blockers including non-selective ones like lithium and more selective ones like tideglusib reduced tau phosphorylation, lowered Aβ levels, and improved memory in AD-transgenic mice (Kramer et al., 2012; Shri et al., 2023). Clinically, the GSK3β inhibitor tideglusib was tested in a Phase II trial in mild-to-moderate AD. While it was well tolerated, it did not significantly improve cognitive outcomes over 6 months (Lovestone et al., 2015). Aside from lithium which some studies associate with a lower incidence of dementia, no other GSK3β inhibitors have advanced far in trials. Therefore, this target remains under investigation for AD (Chen et al., 2022; Haussmann et al., 2021). Altogether, GSK3β′s central role in tau pathology and its broader contributions to neuronal injury make it an appealing therapeutic target, though one that may require careful modulation to avoid side effects (Zhao et al., 2024).

## Pathophysiological role of SIRT1 in Alzheimer’s disease

Sirtuin-1 is an NAD^+^-dependent deacetylase that acts as a master regulator of cellular stress responses and metabolic homeostasis (Mehramiz et al., 2023). In the brain, SIRT1 activity generally promotes neuroprotective “anti-aging” effects (Sarubbo et al., 2018). For example, SIRT1 enhances synaptic plasticity, mitochondrial function, and antioxidant defences while dampening inflammation (Jiao and Gong, 2020). Notably, SIRT1 levels decline with age, and studies have found reduced SIRT1 expression in the brains of AD patients compared to age-matched controls (Fernandez et al., 2024). This loss may contribute to disease progression, as numerous models indicate that boosting SIRT1 function is beneficial in neurodegeneration (Jiao and Gong, 2020; Kim et al., 2007). Accordingly, SIRT1 has emerged as an appealing therapeutic target in AD.

SIRT1 counteracts both major pathological hallmarks of AD (Mishra et al., 2021). First, it mitigates the amyloid pathway by shifting APP processing towards the non-amyloidogenic route, upregulating the α-secretase that precludes Aβ formation and downregulating β-secretase (Zhang and Tang, 2023). In AD mouse models, overexpression of SIRT1 or treatment with SIRT1 activators results in lower Aβ production and plaque burden, as well as enhanced clearance of Aβ peptides (Campagna et al., 2018; Islam et al., 2022). Second, SIRT1 helps control tau pathology by directly deacetylates tau protein, promoting tau degradation via the proteasome, and it indirectly reduces abnormal tau phosphorylation (H. Liu et al., 2022). For instance, mice lacking neuronal SIRT1 show accelerated tau accumulation and cognitive decline, whereas SIRT1 overexpression or activation reduces tau acetylation and slows the spread of tau tangles (Min et al., 2018; Neuroscience, 2023). SIRT1’s influence extends to neuroinflammation and oxidative stress as well (Jiao and Gong, 2020). Active SIRT1 can deacetylate the NF-κB transcription factor, thereby suppressing pro-inflammatory cytokine production, and can activate the transcriptional coactivator PGC-1α to bolster mitochondrial biogenesis and antioxidant gene expression (V. Singh and Ubaid, 2020). Through these multifaceted actions, SIRT1 enhances neuronal survival and resilience.

The protective role of SIRT1 in AD is underscored by pharmacological studies. Small-molecule SIRT1 activators like resveratrol have shown beneficial effects in preclinical models (Islam et al., 2022). For example, resveratrol reduced brain Aβ levels, neuroinflammation, and memory loss in AD mice (Wiciński et al., 2020). In a pilot clinical trial in AD patients, high-dose resveratrol was found to penetrate the blood–brain barrier and modulate AD biomarkers (such as Aβ40 levels and inflammatory markers), supporting the idea that SIRT1 activation is achievable in humans (Turner et al., 2015). Although resveratrol did not significantly improve cognition over 12 months, the trial confirmed target engagement and has spurred interest in more potent or bioavailable SIRT1 activators (Turner et al., 2015). Overall, SIRT1 acts as a crucial endogenous defence factor against AD pathology, and strategies to enhance SIRT1 function are expected to ameliorate multiple toxic cascades in the disease (Ren et al., 2019).

## Mechanistic rationale for dual GSK3β inhibition and SIRT1 activation

**FIGURE 2:**
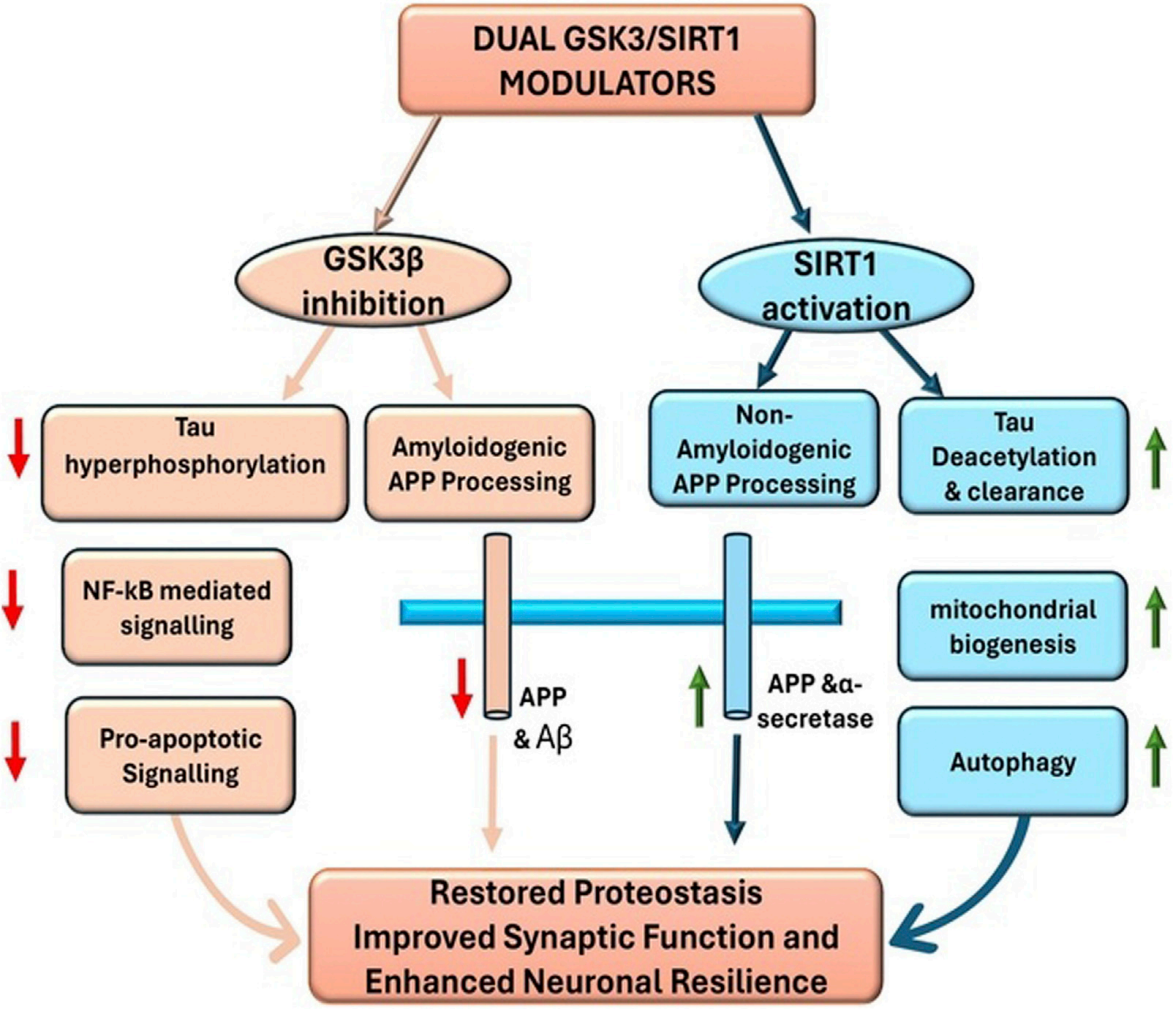
The molecular pathways targeted by dual GSK3β/SIRT1 modulators. Together, GSK3β inhibition suppresses pathological cascades while SIRT1 activation enhances protective mechanisms, converging on improved neuronal survival and resilience.

The appeal of concurrently targeting GSK3β and SIRT1 lies in their largely opposing roles in neuronal health (Figure 2). GSK3β hyperactivity accelerates the molecular damage of AD by phosphorylating tau and promoting Aβ-generating processes, whereas SIRT1 activity facilitates damage control and repair by enhancing clearance of misfolded proteins and bolstering stress defences (Cardoso et al., 2016; Zhao et al., 2024). Breaking this vicious cycle at both points, by inhibiting GSK3β and activating SIRT1, should restore a healthier balance in neurons than addressing either alone. In practical terms, a dual-action molecule would simultaneously reduce tau hyperphosphorylation and proteotoxic stress via GSK3β inhibition while heightening the cell’s proteostasis and survival mechanisms via SIRT1 activation, leading to synergistic neuroprotection.

There may also be metabolic advantages to this dual strategy. AD is often associated with brain insulin resistance and energy deficits, sometimes called “type III diabetes”. (Kciuk et al., 2024) GSK3β inhibition tends to improve insulin signalling since GSK3β is normally inactivated by insulin, and SIRT1 activation mimics aspects of caloric restriction, enhancing mitochondrial function and insulin sensitivity (Cunha-Santos et al., 2016; Kciuk et al., 2024). Thus, a GSK3β/SIRT1 dual-modulator could simultaneously alleviate AD-related metabolic impairments. An added benefit is that engaging both targets in one molecule ensures they act in concert within the same cells and at the same time. This could allow for a lower degree of GSK3β inhibition, limiting side effects of full kinase blockade, while still achieving strong efficacy. In sum, dual modulation addresses both “the brake and the accelerator” of neurodegeneration, curbing a key pathological driver and reinforcing the brain’s own protective pathways.

## Strategies for designing dual-acting GSK3β/SIRT1 compounds

Designing a single compound with two distinct activities (kinase inhibition and deacetylase activation) is a formidable challenge. The two targets, GSK3β and SIRT1, differ greatly in structure and enzymatic mechanism (Pandya et al., 2025; Wang et al., 2019). GSK3β is a kinase with an ATP-binding catalytic pocket (Shri et al., 2023). Inhibitors typically bind in this pocket or at an allosteric site, and most known GSK3β inhibitors are small planar heterocycles that mimic ATP or its transition state (Shri et al., 2023). SIRT1, on the other hand, is a protein that catalyses NAD^+^-dependent deacetylation (Khan et al., 2021). Uniquely, SIRT1 activators are not active site binders; instead, known SIRT1 activators like resveratrol bind to an allosteric site on SIRT1 that is associated with the enzyme’s substrate-binding domain (Liu et al., 2023). Thus, a dual-acting compound must ideally have two functional components: one that can fit into GSK3β′s ATP site to inhibit its activity, and another that can interact with SIRT1’s activation site to enhance its activity. Several drug design strategies can be employed to create such dual-function molecules:

Pharmacophore merging: Identification of key pharmacophoric features required for each target and attempt to integrate them into a single scaffold. This approach is conceptually straightforward but often difficult in practice, given the very different structural requirements of a kinase’s ATP site versus SIRT1’s allosteric activation site.

Linker-based hybridization: A more practical strategy is to chemically link two distinct pharmacophores, one known to inhibit GSK3β and one known to activate SIRT1, with an appropriate spacer. The resulting hybrid molecule essentially has two functional “heads” connected by a tether. For example, a tacrine–pyrimidone hybrid designed to target AChE and GSK3β demonstrated the feasibility of this approach by showing cognitive benefits in animal models (Yao et al., 2021). Similarly, a dual GSK3β/SIRT1 hybrid would require optimizing the linker length and flexibility so that both moieties can engage their targets simultaneously.

Fragment fusion and structure-based design: Using computational modelling and fragment-based drug design, one can identify smaller substructures that bind to each target and then tether or fuse them into a larger molecule (de Esch et al., 2022). Virtual screening methods can also search for “privileged” scaffolds capable of interacting with both GSK3β and SIRT1 (Grotsch et al., 2024). Any hits can be optimized via medicinal chemistry approaches (Hoffer et al., 2018). This strategy relies heavily on X-ray crystal structures and molecular docking simulations to ensure the fused compound can adopt conformations that fit both binding sites.

Cleavable bi-functional prodrugs: Another tactic is to join two separate drugs (a GSK3β inhibitor and a SIRT1 activator) with a cleavable linker into a single conjugate. This conjugate essentially a prodrug (Kratz et al., 2008), can cross the blood–brain barrier as one unit, then be metabolized in the brain to release the two active agents together. While this yields two molecules *in vivo* (so it is not a true single-entity drug), it guarantees concurrent delivery of both functional components to the target tissue.

Targeting upstream pathways: Instead of directly hitting GSK3β and SIRT1, a single compound could be designed to modulate an upstream regulator that in turn affects both. For instance, activation of AMP-activated protein kinase (AMPK) in neurons will indirectly inhibit GSK3β via Akt signalling and increase SIRT1 activity by boosting NAD^+^ levels (Zhao et al., 2024). A compound that activates AMPK in the brain such as certain plant molecule like berberine, thus produces the functional effect of GSK3β inhibition plus SIRT1 activation without binding target directly (Qin et al., 2020).

Natural compound-inspired design: Many natural compounds already exhibit multi-target effects relevant to GSK3β and SIRT1. Resveratrol and berberine, for example, both activate SIRT1 and have been reported to suppress GSK3β activity indirectly (Zhang and Tang, 2023). Medicinal chemists often use such polyfunctional molecules as starting templates, modifying their structures to improve potency, specificity, or pharmacokinetic properties while retaining dual activity. The goal is to capture the “privileged” multi-target pharmacophore that evolutionarily derived compounds like these possess.

## Dual-acting compounds and preclinical evidence

While the concept of a dual GSK3β inhibitor/SIRT1 activator is relatively new, several compounds, naturally derived substances have been identified that exhibit the desired dual activity profile either directly or indirectly. Notable examples, including resveratrol, berberine, quercetin and their effects in preclinical AD models are summarized below.

## Resveratrol

**FIGURE 3:**
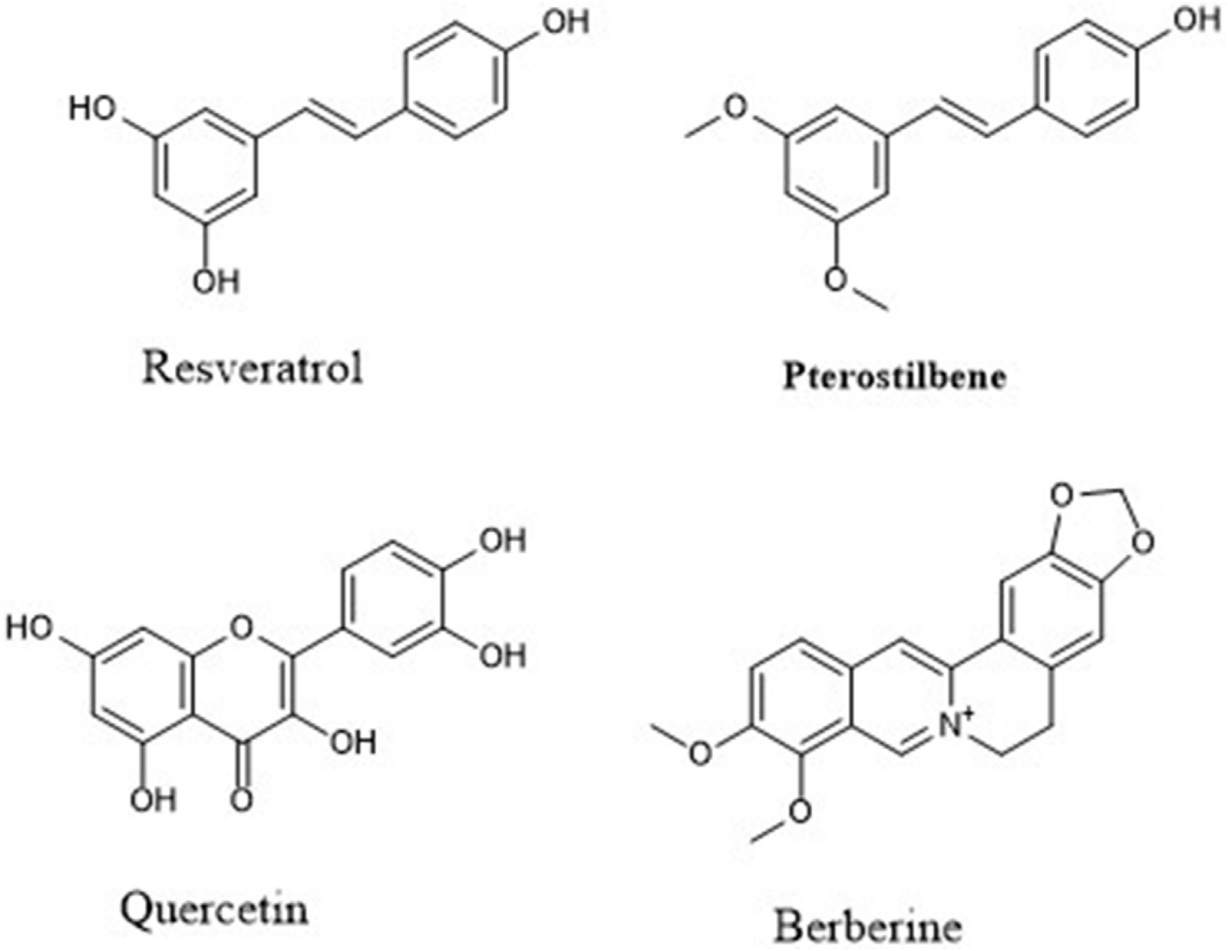
Structures of dual acting GSK-3β inhibitors and SIRT1 activators.

A well-known SIRT1 activator (Figure 3) has also been demonstrated to inhibit GSK-3β indirectly through activation of the AKT signalling pathway, contributing to its neuroprotective profile in ischemic animal models (Park et al., 2019). Resveratrol increases SIRT1 activity and triggers downstream pathways (e.g., AMPK/Akt) that lead to inhibitory phosphorylation of GSK3β (Zhang and Tang, 2023). In AD models, resveratrol has demonstrated robust neuroprotective effects. In the 5xFAD (Tg6799) mouse model, oral administration of resveratrol (∼60 mg/kg daily for 60 days) significantly reduced amyloid plaque deposition, lowered Aβ_42_ levels, and improved spatial working memory in Y-maze and Morris water maze tasks (Chen et al., 2019). In the PS19 tauopathy model, 5-week resveratrol treatment in 6-month-old mice rescued cognitive deficits, attenuated tau pathology (phosphorylated tau and oligomers), suppressed neuroinflammation (glial activation and pro-inflammatory cytokines), and preserved synaptic markers in hippocampal region (Sun et al., 2019). Similarly, a 52-week clinical trial in patients with mild AD, high-dose resveratrol was found to be safe and penetrant to the brain, and it stabilized certain biomarkers (such as Aβ40 levels) relative to placebo, although cognitive outcomes did not significantly improve in that timeframe (Turner et al., 2015).

## Quercetin

A bioactive flavonoid (Figure 3) found in fruits and vegetables. Quercetin has been reported to enhance SIRT1 activity and inhibit GSK3β in a concentration-dependent manner (Ungurianu et al., 2024). In a rat model of AD induced by Aβ injection, oral quercetin at 40 mg/kg/day for 1 month improved learning and memory (passive avoidance, MWM), and enhanced adult hippocampal neurogenesis by increasing progenitor proliferation and DCX/BrdU-NeuN positive cells in the dentate gyrus. It also upregulated neurotrophic and transcriptional regulators (BDNF, NGF, CREB, EGR-1), suggesting a mechanism involving enhanced neurogenesis rather than direct Aβ/tau clearance. In hippocampal neuronal models, quercetin has been shown to exert neuroprotective effects by inhibiting GSK3β signalling, thereby attenuating tau hyperphosphorylation and oxidative stress. In HT22 cells, quercetin pre-treatment reduced okadaic acid–induced tau phosphorylation (Ser199, Ser396, Thr205, Thr231) and oxidative damage via modulation of the PI3K/Akt/GSK3β, MAPK, and NF-κB pathways (Jiang et al., 2016).

## Pterostilbene

Pterostilbene (Figure 3), a natural stilbene related to resveratrol, exerts anti-inflammatory and neuroprotective effects that involve SIRT1 activation and modulation of MAPK (including p38) signalling (Jayakumar et al., 2021; Zhu et al., 2022). It has also been shown to reduce GSK-3β activity and attenuate tau hyperphosphorylation and Aβ accumulation in rodent brain/hippocampal tissue (Li et al., 2022). In STZ-induced C57BL/6J mice, daily oral pterostilbene (20 mg/kg, 5 weeks) improved Morris water maze performance and reduced hippocampal Aβ1–42 accumulation, tau hyperphosphorylation, oxidative stress, and inflammation, partly via downregulation of MAOB (Li et al., 2022). Similarly, Pterostilbene (40 mg/kg) improved memory and neuronal plasticity in Aβ25–35 AD models, acting via SIRT1/Nrf2-mediated antioxidant effects and inhibition of mitochondrial apoptosis (Zhu et al., 2022). Its improved pharmacokinetic profile relative to resveratrol, including enhanced bioavailability and CNS penetration, makes it a candidate of interest for further translational studies.

## Berberine

Berberine (Figure 3) has been shown to activate the AMPK–SIRT1–PGC-1α axis, enhancing mitochondrial resilience in aging models (Yu et al., 2018). In APP/PS1 transgenic mice, berberine alleviated cognitive deficits and reduced both tau phosphorylation and Aβ42 production by suppressing endoplasmic reticulum stress. Mechanistically, berberine decreased GSK3β activity, lowering tau hyperphosphorylation, and inhibited the PERK/eIF2α/BACE1 signalling pathway, thereby reducing Aβ42 generation (Wu et al., 2021).

## Selenium

Selenium (Se) has been shown to modulate the AKT/GSK-3β signaling axis and enhance SIRT1-mediated mechanisms, elevating antioxidant defenses (SOD2, GPx, GSH) and attenuating pro-apoptotic signalling in cardiac hypertrophy models (Shengyu et al., 2022). In 3×Tg-AD mice, low-dose treatment with three selenium compounds—Se-methylselenocysteine (SMC), selenomethionine (SeM), and sodium selenate (SeNa)—enhanced brain selenium levels, boosted antioxidant capacity, regulated amino acid metabolism, and improved cognition by alleviating synaptic deficits. Mechanistically, SMC increased thioredoxin reductase and reduced tau phosphorylation via GSK-3β inhibition (Zhang et al., 2023).

## 2D structural similarity analysis of dual-acting natural compounds

**FIGURE 4:**
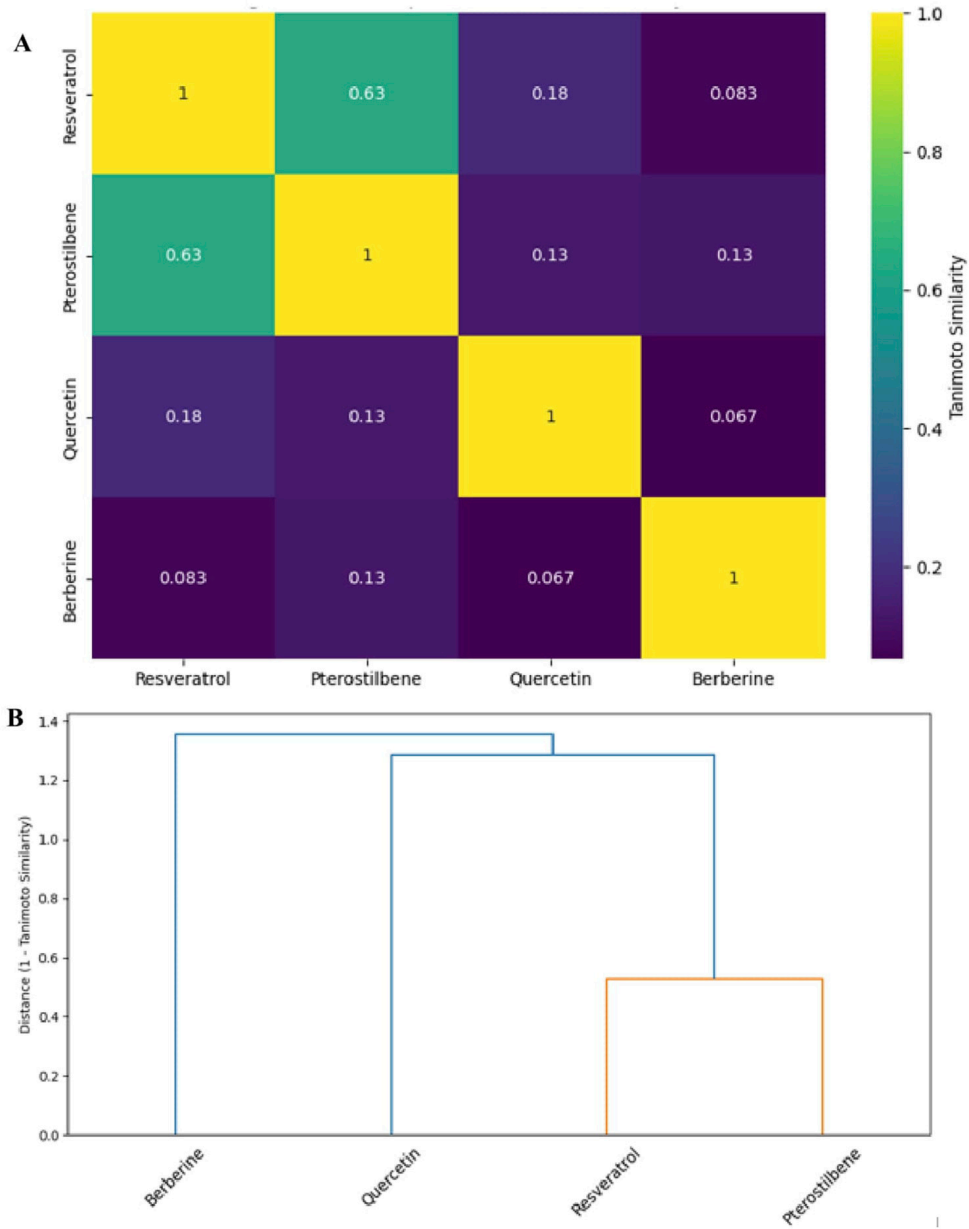
**(A)** Heatmap of pairwise 2D similarity (Tanimoto coefficients) among the natural compounds. Brighter colours (yellow/green) indicate higher similarity, and darker colours (blue/purple) indicate lower similarity. Each cell is annotated with the similarity value for clarity. **(B)** Hierarchical clustering (dendrogram) of the compounds based on 2D structural similarity. The horizontal axis represents the distance (1 – Tanimoto similarity); shorter linkage distance indicates higher structural resemblance. Resveratrol and pterostilbene form a tight cluster (orange branch), highlighting their shared stilbene scaffold, while quercetin and especially berberine are more structurally divergent.

To quantify structural relatedness among key natural compounds with dual GSK3β inhibition and SIRT1 activation, molecular structures of identified natural compounds were encoded using Morgan circular fingerprints (also known as ECFP4) generated via the RDKit cheminformatics toolkit (Landrum, 2019). Pairwise 2D similarity analysis was performed using the Tanimoto coefficient based on Morgan circular fingerprints (Rogers and Hahn, 2010). These fingerprints encode atom environments up to a defined radius (radius = 2, 2048 bits), and are widely used for cheminformatics-based similarity assessments (Todeschini and Consonni, 2009). The similarity matrix (Table 1) shows how chemically close these compounds are in terms of structural fingerprints (ranges from 0 to 1). Similarity matrices were constructed using pairwise Tanimoto coefficients, and clustering was performed using hierarchical average linkage on the distance matrix (1 – Tanimoto) (Figure 4).

**TABLE 1:** Pairwise Tanimoto similarity matrix for selected dual-acting compounds (Landrum, 2019; Rogers and Hahn, 2010).

Compound	Resveratrol	Pterostilbene	Quercetin	Berberine
Resveratrol	1.00	0.63	0.18	0.08
Pterostilbene	0.63	1.00	0.13	0.13
Quercetin	0.18	0.13	1.00	0.07
Berberine	0.08	0.13	0.07	1.00

Resveratrol and pterostilbene share a high structural similarity (Tanimoto coefficient = 0.63), as both are stilbenes characterized by a C6–C2–C6 skeleton. Pterostilbene is a dimethoxylated analogue of resveratrol, a modification that reduces polarity and enhances bioavailability (Socała et al., 2024; Wiciński et al., 2020). In contrast, quercetin, and berberine exhibit low similarity to the stilbenes (Tanimoto <0.20) and to each other, underscoring their membership in distinct chemical families. Nevertheless, despite the overall low similarity, all compounds possess shared structural features such as aromatic ring systems, π-conjugation, and functional groups (e.g., hydroxyl and methoxy substituents) that facilitate hydrogen bonding and π–π interactions with biological targets.

## Scaffold analysis and implications for rational drug design

The stilbene scaffold, exemplified by resveratrol and pterostilbene, emerges as a privileged structure for dual GSK3β/SIRT1 modulation and is well-suited for pharmacophore-based expansion strategies (Socała et al., 2024; Zhang and Tang, 2023). Quercetin provides a flavonoid-based alternative, distinguished by multiple hydroxyl groups and notable antioxidant capacity (Singh et al., 2021). Although structurally distinct from the stilbenes, it modulates both targets through complementary biochemical pathways (Ungurianu et al., 2024). Berberine, an alkaloid with a rigid polycyclic framework, achieves dual activity primarily via indirect activation of the AMPK pathway (Qin et al., 2020). This illustrates that functional similarity does not necessarily correlate with structural similarity. These trends suggest that future hybrid molecules could integrate phenolic or stilbene-derived pharmacophores into heterocyclic scaffolds like berberine to enhance synergistic target engagement, improve central nervous system permeability, and optimize pharmacokinetic profiles.

## Computational approaches in dual-target drug discovery

**FIGURE 5:**
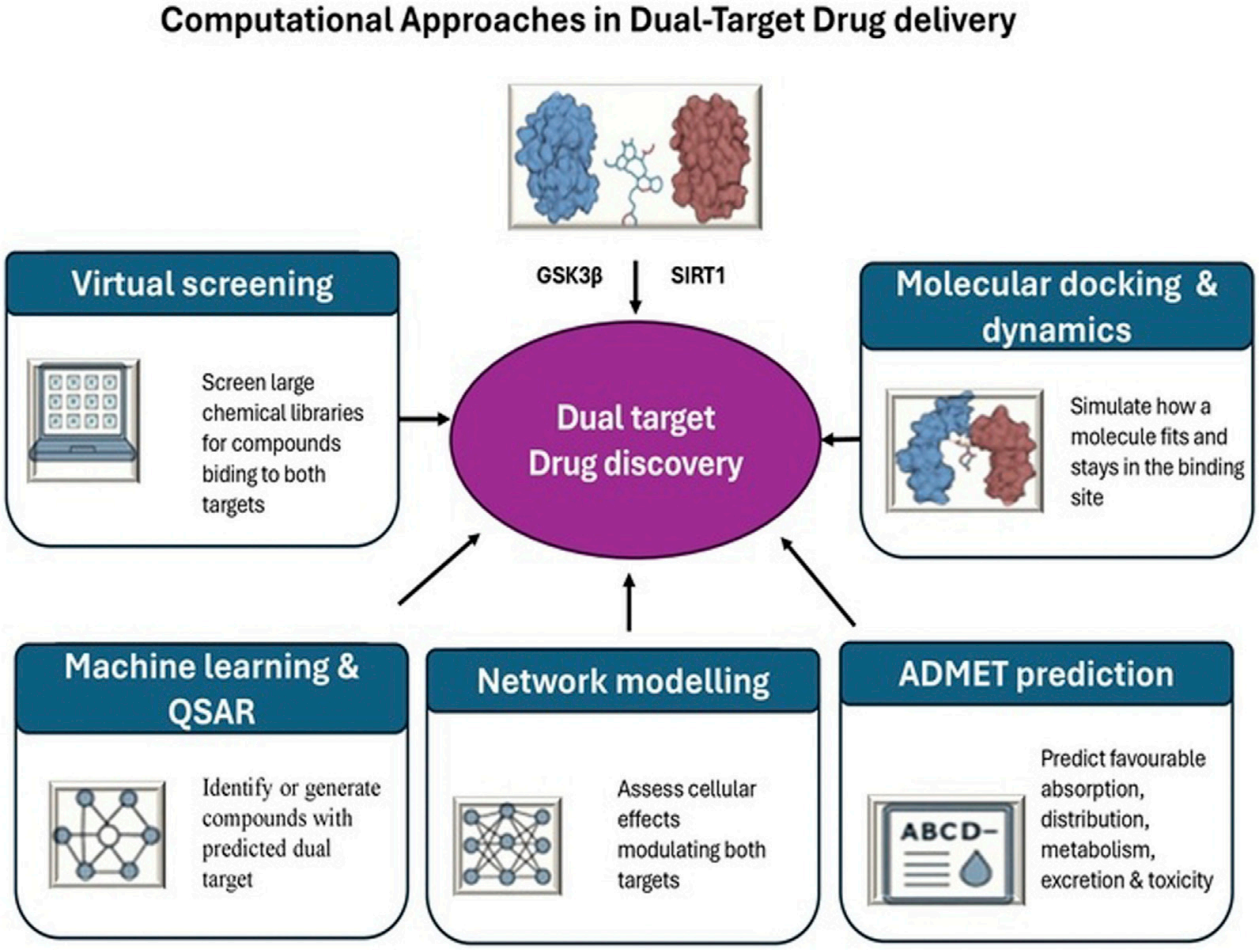
Computational approaches in dual-target drug discovery.

Computational approaches (Figure 5) remain vital tools for implementing the “fail fast, fail cheap” strategy in drug discovery. Key *in silico* methods include:

Structure based virtual screening: Screening large chemical libraries *in silico* against the structures of GSK3β and SIRT1 can identify candidate molecules that bind to each target. Researchers then look for overlapping hits or design compounds predicted to bind both proteins. This method efficiently narrows down multi-target lead compounds for synthesis and testing.

Molecular docking and dynamics: For rationally designed hybrids, computational docking simulations model how one part of a molecule fits into GSK3β′s active site and how the other part might interact with SIRT1. Molecular dynamics simulations help verify that a single compound can adopt conformations to engage both targets without steric clashes (Bera and Payghan, 2019). These tools guide optimization of linker length and geometry in dual compounds before they are made.

Machine learning and QSAR: Data-driven approaches assist in predicting dual-target activity (Feng et al., 2024). Quantitative structure–activity relationship (QSAR) models and modern AI techniques can be trained on known GSK3β inhibitors and SIRT1 activators to forecast which molecular features confer activity at both. Some generative algorithms have even proposed novel scaffolds optimized for simultaneous binding, giving chemists new ideas to synthesize (Grisoni et al., 2021).

Network pharmacology modelling: Computational models of cellular signalling and gene networks help predict system-level effects of inhibiting GSK3β and activating SIRT1 together (Zheng et al., 2021). These simulations can highlight synergistic outcomes or potential conflicts by mapping how dual modulation influences downstream pathways (Wnt/β-catenin, insulin/Akt, FOXO, etc.). Network models thus reinforce the rationale, often showing that dual intervention stabilizes cellular homeostasis more robustly than single-target changes (Tang and Aittokallio, 2014).

ADMET property prediction: Because dual-acting molecules can be larger or more complex, early *in silico* filtering for favourable absorption, distribution, metabolism, and excretion (ADMET) properties is crucial. Computational tools predict blood–brain barrier permeability, metabolic stability, and potential off-target toxicity of candidate compounds (Daina et al., 2017). This helps prioritize which dual-action candidates are most likely to be viable drugs before committing to labour-intensive synthesis.

## Challenges in dual-target drug development

Developing dual-target drugs that simultaneously modulate GSK3β and SIRT1 presents a number of scientific and translational challenges. From a medicinal chemistry standpoint, designing a single molecule capable of engaging two mechanistically distinct targets often results in increased structural complexity, which may compromise potency, specificity, or drug-like properties (Benek et al., 2020; Singh et al., 2016). Achieving the necessary pharmacophore balance requires extensive iterative optimization to ensure that both the GSK3β-inhibitory and SIRT1-activating functionalities remain effective without interfering with one another. In addition, dual-acting compounds are frequently associated with suboptimal pharmacokinetics; increased molecular weight and polarity may reduce oral bioavailability, impede central nervous system (CNS) penetration, or increase susceptibility to metabolic degradation and efflux transporters such as P-glycoprotein (Daina et al., 2017; Socała et al., 2024). These limitations pose significant barriers to achieving therapeutically relevant brain concentrations (Salave et al., 2023). Beyond pharmacokinetics, the risk of off-target interactions also increases with multi-target designs (Hossain and Hussain, 2025). For instance, promiscuity is common among kinase inhibitors (Shri et al., 2023), while polyphenolic SIRT1 activators are known to influence a broad array of cellular targets (Ungurianu et al., 2024). As such, rigorous selectivity profiling and structural refinement are essential to minimize adverse effects (de Esch et al., 2022). Importantly, even on-target effects may be problematic if not appropriately modulated (Rudmann, 2013). GSK3β plays critical roles in glycogen metabolism, neurogenesis, and cell-cycle regulation, and its chronic inhibition may lead to metabolic or proliferative disturbances (Haussmann et al., 2021; Lai et al., 2023). Similarly, excessive activation of SIRT1 may disrupt acetylation-dependent gene regulation or cause metabolic imbalances (Mehramiz et al., 2023). Therefore, it is imperative to establish a therapeutic window that allows for safe and sustained modulation of both targets (Min et al., 2018; Sayas and Ávila, 2021). Establishing that both targets contribute to therapeutic benefit, such as evidence of reduced tau phosphorylation (GSK3β) and improved metabolic or synaptic biomarkers (SIRT1), may be required by regulatory agencies (Lista et al., 2024). Finally, the development and approval process for polypharmacological drugs is inherently more complex than for single-target agents (Kabir and Muth, 2022). Optimization is multidimensional, intellectual property issues may arise when incorporating known pharmacophores, and regulatory pathways may require demonstration of added value over monotherapies (Prajapati et al., 2024). Despite these hurdles, the potential to achieve robust, disease-modifying efficacy justifies the increased effort and underscores the promise of dual GSK3β/SIRT1 targeting strategies in Alzheimer’s disease (Benek et al., 2020; Grisoni et al., 2021).

## Nanoformulation strategies for enhancing clinical translation for dual GSK3β/SIRT1 modulators

Despite promising preclinical evidence, natural compounds such as resveratrol, quercetin, and berberine have not advanced into clinical use for Alzheimer’s disease, largely due to poor solubility, rapid metabolism, and low blood–brain barrier (BBB) penetration (Rahman et al., 2021; Zivari-Ghader et al., 2024). Addressing these pharmacokinetic and pharmacodynamic barriers is crucial to harnessing their full therapeutic potential in Alzheimer’s disease. Nanoparticle-based systems represent a promising approach to improve the delivery of such dual modulators. Polymeric nanoparticles (e.g., PLGA or chitosan-based systems) can encapsulate polyphenols, improving stability, prolonging systemic circulation, and enabling controlled release (Elmowafy et al., 2023). Solid lipid nanoparticles (SLNs) and nanostructured lipid carriers (NLCs) have demonstrated improved brain targeting and bioavailability of resveratrol and curcumin compared with free compounds (Elmowafy et al., 2023; Umerska et al., 2018). Similarly, liposomes can enhance aqueous solubility and protect bioactive molecules from enzymatic degradation, thereby facilitating greater CNS delivery (Wang et al., 2019). Moreover, targeted nanoparticles decorated with ligands such as transferrin, lactoferrin, or ApoE-mimetic peptides can exploit receptor-mediated transcytosis to improve CNS selectivity (Kato et al., 2023; Neves et al., 2016; Youssef et al., 2025), making them particularly relevant for dual GSK3β/SIRT1 modulation. Thus, nanotechnology-enabled strategies may overcome pharmacokinetic limitations and accelerate the translation of dual GSK3β/SIRT1 modulators into clinical application.

## Discussion and future perspectives

The mechanistic and therapeutic rationale for dual modulation of GSK3β and SIRT1 in Alzheimer’s disease (AD) represents an evolution in the strategic design of disease-modifying agents. This review synthesizes current evidence supporting the feasibility and promise of targeting these two functionally complementary proteins: GSK3β, a pro-pathogenic kinase implicated in tau hyperphosphorylation and neuroinflammation, and SIRT1, a neuroprotective deacetylase that enhances proteostasis, anti-inflammatory responses, and mitochondrial function (Chen et al., 2024; Song et al., 2022). Preclinical studies consistently demonstrate that inhibiting GSK3β reduces tau pathology and Aβ generation, while activating SIRT1 promotes non-amyloidogenic APP processing and enhances the clearance of neurotoxic species (Mehramiz et al., 2023; Sayas and Ávila, 2021). Breaking this pathogenic circuit through concurrent modulation offers a more holistic intervention than either target alone.

In this context, dual-acting compounds embody the principles of rational polypharmacology (Kabir and Muth, 2022). The failure of single-target therapies in late-stage clinical trials, despite effective engagement of their molecular targets (Jain et al., 2023), underscores the inadequacy of linear approaches in a multifactorial disease like AD. By simultaneously curbing a key driver of neurodegeneration (GSK3β) and enhancing an intrinsic defence system (SIRT1), dual-target compounds promise additive or synergistic effects that could translate to superior neuroprotection. This approach aligns with current literature on Multi-target directed ligands (MTDLs), where compounds designed to influence several disease-relevant mechanisms have shown improved efficacy in preclinical models (Zhang et al., 2024). Examples include tacrine–pyrimidone, IQ6 (SSN) and thiosemicarbazone–acridine hybrids that outperform mono-target comparators (Khan et al., 2025; Sousa et al., 2024; Yao et al., 2021). While no GSK3β/SIRT1 dual modulators have yet been approved for Alzheimer’s disease, approved dual-acting drugs in other therapeutic areas such as carvedilol (β-/α-blocker) (Frishman, 1998), lapatinib (EGFR/HER2 inhibitor) (Tsang et al., 2011), tirzepatide (GIP/GLP-1 agonist) (Fisman and Tenenbaum, 2021), and romosozumab (sclerostin antibody) (Miller et al., 2021) provide translational precedent. These cases reinforce the feasibility and therapeutic value of dual-target pharmacology in complex diseases, supporting the rationale for pursuing GSK3β/SIRT1 co-modulators as a template for a new generation of AD therapeutics aimed at restoring systems-level homeostasis in the aging brain.

Nevertheless, translating this paradigm into clinically viable compounds presents substantial challenges. The divergent structural and mechanistic nature of GSK3β and SIRT1 complicates the design of single molecules with dual activity. Strategies such as linker-based hybridization, fragment fusion, or natural-product-inspired scaffolding must overcome issues of molecular size, polarity, and conformational flexibility to ensure blood–brain barrier (BBB) penetration and oral bioavailability (Alarcón-Espósito et al., 2021). Furthermore, careful tuning of pharmacodynamics is essential. Excessive GSK3β inhibition can disrupt vital cellular processes including neurogenesis and glucose metabolism, while chronic SIRT1 activation may lead to dysregulated gene expression (Wang et al., 2022; Yang et al., 2022). Selectivity profiling, structure–activity optimization, and metabolic stability studies will be critical to ensure a favourable therapeutic window (Cerny et al., 2020; Huggins et al., 2012; Wang et al., 2015).

Despite these challenges, several promising avenues are emerging. Natural compounds such as resveratrol, pterostilbene, berberine, and quercetin exemplify polyfunctional scaffolds capable of indirectly modulating both GSK3β and SIRT1, albeit with modest potency (Khezri et al., 2024; Qu et al., 2023; Ungurianu et al., 2024; Zhang and Tang, 2023). Their structural diversity and pharmacological profiles offer a foundation for scaffold-hopping and structure-guided optimization. Computational methods, including virtual screening, molecular dynamics, and AI-driven generative design can further accelerate lead identification and scaffold refinement (Son et al., 2024). Notably, 2D similarity and clustering analyses presented in this review indicate that stilbenes such as resveratrol and pterostilbene represent a privileged scaffold class for dual-target engagement (Landrum, 2019; Rogers and Hahn, 2010). Hybrid design strategies that integrate such motifs into novel heterocyclic frameworks could yield compounds with improved potency, CNS penetration, and drug-likeness.

Future research should also prioritize translational readiness. Rigorous *in vivo* studies must confirm target engagement and assess pharmacodynamic biomarkers such as tau phosphorylation, Aβ clearance, and mitochondrial function (Cummings et al., 2025). Advanced delivery systems including CNS-specific prodrugs, brain-penetrant nanoparticles, and conjugates may improve bioavailability and spatial selectivity (Kratz et al., 2008). In addition, gene therapy platforms that modulate GSK3β and SIRT1 expression in a cell-specific manner offer a long-term therapeutic strategy, although one still in early development (Yu et al., 2023). From a clinical trial perspective, adaptive designs and stratified cohorts based on biomarker profiles (e.g., insulin resistance, tau burden) could enhance sensitivity to detect cognitive or functional benefits of dual-acting agents (Cummings et al., 2020). Moreover, as preclinical evidence implicates GSK3β and SIRT1 dysregulation in other neurodegenerative diseases, such as Parkinson’s and Huntington’s (D’Mello, 2021; Lloret and Beal, 2019), dual-target compounds may have utility beyond AD, broadening their therapeutic scope.

In conclusion, Alzheimer’s disease, characterized by its multifactorial etiology and complex interplay of pathological processes (Samanta et al., 2022), necessitates therapeutic strategies that are similarly multifaceted. Dual-acting compounds that simultaneously inhibit GSK3β and activate SIRT1 exemplify a rational polypharmacological approach, addressing pathogenic drivers such as tau hyperphosphorylation, neuroinflammation, and synaptic dysfunction, while reinforcing protective mechanisms including autophagy, mitochondrial resilience, and metabolic regulation. This dual modulation may synergistically slow disease progression by suppressing deleterious cascades and enhancing intrinsic cellular defences. Substantial challenges remain in optimizing dual scaffolds for drug-likeness, selectivity, and brain penetrance, and in validating efficacy clinically. Natural compounds such as resveratrol and quercetin illustrate proof of concept but face limitations of poor solubility and bioavailability. Continued innovation in medicinal chemistry, together with pharmacokinetic optimization including nanoformulation and nanocarrier delivery, and pharmacodynamic evaluation will be critical to overcoming these hurdles. With such advances, dual GSK3β/SIRT1 modulators may emerge as next-generation disease-modifying agents capable of restoring network homeostasis in the aging brain, underscoring the value of a systems-oriented therapeutic framework rather than targeting isolated nodes.
